# An Automated Flow for Directed Evolution Based on Detection of Promiscuous Scaffolds Using Spatial and Electrostatic Properties of Catalytic Residues

**DOI:** 10.1371/journal.pone.0040408

**Published:** 2012-07-11

**Authors:** Sandeep Chakraborty

**Affiliations:** Department of Biological Sciences, Tata Institute of Fundamental Research, Mumbai, India; University of South Florida College of Medicine, United States of America

## Abstract

The aspiration to mimic and accelerate natural evolution has fueled interest in directed evolution experiments, which endow or enhance functionality in enzymes. Barring a few de novo approaches, most methods take a template protein having the desired activity, known active site residues and structure, and proceed to select a target protein which has a pre-existing scaffold congruent to the template motif. Previously, we have established a computational method (CLASP) based on spatial and electrostatic properties to detect active sites, and a method to quantify promiscuity in proteins. We exploit the prospect of promiscuous active sites to serve as the starting point for directed evolution and present a method to select a target protein which possesses a significant partial match with the template scaffold (DECAAF). A library of partial motifs, constructed from the active site residues of the template protein, is used to rank a set of target proteins based on maximal significant matches with the partial motifs, and cull out the best candidate from the reduced set as the target protein. Considering the scenario where this ‘incubator’ protein lacks activity, we identify mutations in the target protein that will mirror the template motif by superimposing the target and template protein based on the partial match**.** Using this superimposition technique, we analyzed the less than expected gain of activity achieved by an attempt to induce β-lactamase activity in a penicillin binding protein (PBP) (PBP-A from *T. elongatus*), and attributed this to steric hindrance from neighboring residues. We also propose mutations in PBP-5 from *E. coli*, which does not have similar steric constraints**.** The flow details have been worked out in an example which aims to select a substitute protein for human neutrophil elastase, preferably related to grapevines, in a chimeric anti-microbial enzyme which bolsters the innate immune defense system of grapevines.

## Introduction

Directed evolution experiments aspire to mimic and accelerate natural evolution. The methodology typically consists of random mutations [1], in vitro recombination [2]–[6], continuous evolution [7], [8], intramolecular relocation of the N and C termini of a protein [9], [10] and high-throughput screening [11], [12]. While a few exceptional de novo approaches that endow function in proteins from scratch have been successful [13]–[17], most techniques target residues in the catalytic site or in its close vicinity for mutational purposes [18]–[20]. Directed evolution techniques have also sought out the remnants of secondary activities [21]–[23], with the rationale that the presence of a pre-existing catalytic scaffold increases the odds of success in endowing the activity to the protein [24]–[28].

Previous work by our group has established a computational method (CLASP) for the detection of active sites [29]. CLASP analysis of the catalytic residues in Class A β-lactamases identified the dichotomy in the proton abstraction mechanism [30], Based on this idea, we proposed a method that enumerates the possible pathways for proton abstraction [31]. We also outlined a computational methodology to quantify promiscuous activities in a wide range of proteins [32]. Jensen hypothesized that ancient enzymes were sparse and promiscuous [33], and this promiscuity formed the basis of the evolution of complex organisms through gene duplication and specialization [34], [35]. This hypothesis germinated the idea of selecting promiscuous active sites to serve as a pre-existing scaffold for directed evolution.

In the current work, we present a computational method that selects a protein which has a significant match with a desired catalytic scaffold - *D*irected *e*volution using *C*LASP: *a*n *a*utomated *f*low (DECAAF).

**Figure 1:**
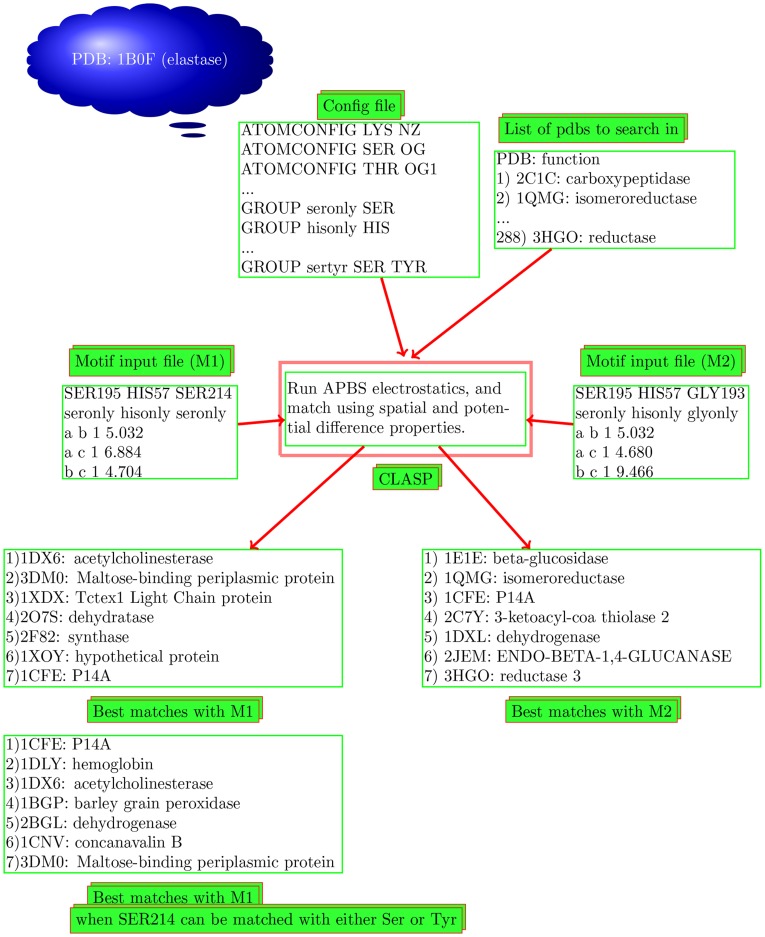
DECAAF flow to select a plant protein with a significant presence of an elastase-like scaffold.

We create a library of partial motifs from the template scaffold and rank a set of proteins based on maximal significant matches with this library. Manual screening, considering further substantiating evidence, is used at this stage to cull the best candidate from the reduced set of proteins with significant congruence. For example, a particular protein would be more suitable if related proteins in the same superfamily are known to have the function under consideration.

The second part of the flow addresses the problem of predicting mutations that mirrors catalytic properties (efficiency, substrate specificity, etc.) of the template protein. In order to replicate the template scaffold in the target protein, we first superimpose the template and target proteins based on the partial match. The residues in the target protein that are spatially close to the residues in the template motif, if they exist and are not part of the partial motif, are enumerated as possible candidates for site directed mutagenesis techniques to build on [18]–[20]. In contrast to the structural homology used by DECAAF, a recent method uses phylogenetic and sequence analyses to predict such amino acid substitutions [36].

**Table 1:** DECAAF SCORES (DScore) - best matches for motifs of length 3: The best matching protein (PDBid:1DX6) is *from Torpedo californica* (Pacific electric ray), an organism not related to plants.

PDB	Description	Dscore
1DX6	acetylcholinesterase	0.048
1CFE	pathogenesis-related protein P14A	0.06
1QMG	acetohydroxy-acid isomeroreductase	0.068
2EFJ	3,7-dimethylxanthine methyltransferase	0.069
1W1O	cytokinin dehydrogenase 1	0.076
3DM0	maltose-binding periplasmic protein	0.077
1E1E	beta-glucosidase	0.077
1AIR	pectate lyase c	0.084
2V6G	progesterone 5-beta-reductase	0.084
3HGO	12-oxophytodienoate reductase 3	0.087

The acetylcholinesterase protein figures in the set of proteins being queried since it has a plant alkaloid galanthamine bound in active site, and we used a keyword search for ‘plant’ in http://www.pdb.org/to obtain the query set of 288 proteins. Hence, the best match was with the P14A protein from Solanum lycopersicum (tomato).

The evolution of serine β-lactamases (Blase) (divided into Classes A, C and D) and the related penicillin binding proteins (PBP) has fascinated and confounded researchers for decades [37]–[39]. The physiological relevance of these proteins remains enigmatic [40], [41]. Surprisingly, few directed evolution experiments have reported success in enhancing deacylation in PBPs [42], [43], the catalytic step that Blases use to hydrolyze β-lactams [44]. Even when successful, the activity in these PBP mutants remains much lower than the Blases (110-fold in [42] and 90-fold in [43]). Sequence alignment identified L158E as a potential mutation that would modify the active site in PBP-A from *T. elongatus* to replicate the active site of Class A Blases, but reported only a 90-fold enhancement in the activity [43]. Firstly, CLASP analysis revealed that the L158E mutant indeed gains potential congruence with the Class A Blase as compared to the wildtype PBP. Subsequently, we demonstrated that Pro159 and Asp160 hinders the approach of substrate, using our superimposition technique. This probably explains the ‘puzzling’ lack of significant improvement in spite of the fact that ‘in the L158E mutant, all the catalytic residues and the interactions characteristic of β-lactamases seem to be present’ [43]. Finally, we chose another PBP-5 from *E. Coli* [45], which is also known to have a similar loop present in Class A Blases and PBP-A from *T. elongatus* [43], [46], and propose that a L153E mutant of this protein can gain significant β-lactamase activity, as the side chain resembles Class A Blase to a greater extent.

**Table 2:** Expanding the set of residues that can match a single position in the input motif.

	Predicted residues			Pairwise Distances in Å			Pairwise Potential difference			
PDB:	a	b	c	ab	ac	bc	ab	ac	bc	Score
1B0F	Ser195	His57	Ser214	5	6.8	4.7	53.7	105.7	51.9	0
1CFE (Ser214 can be Ser only)	Ser49	His48	Ser120	0.1	−0.2	1.9	15.3	83.9	68.6	0.111
1CFE (Ser214 can be Ser or Tyr)	Ser49	His48	Tyr36	0.1	0.3	−0.3	15.3	88.6	73.3	0.023

When the position Ser214 in the input motif (Ser195, His57, Ser214) can be matched by a Ser or Tyr, Tyr36 has a much better spatial orientation with respect to Ser49 and His48 as compared to Ser120 in the P14A protein (PDBid:1CFE). The distances are specified in the reference protein (PDBid:1B0F) in A. For the remaining, we show the deviation from the reference value. Potential differences are in units of kT/e (k is Boltzmann’s constant, T is the temperature in K and e is the charge of an electron). They are absolute values and not shown as deviations.

**Figure 2:**
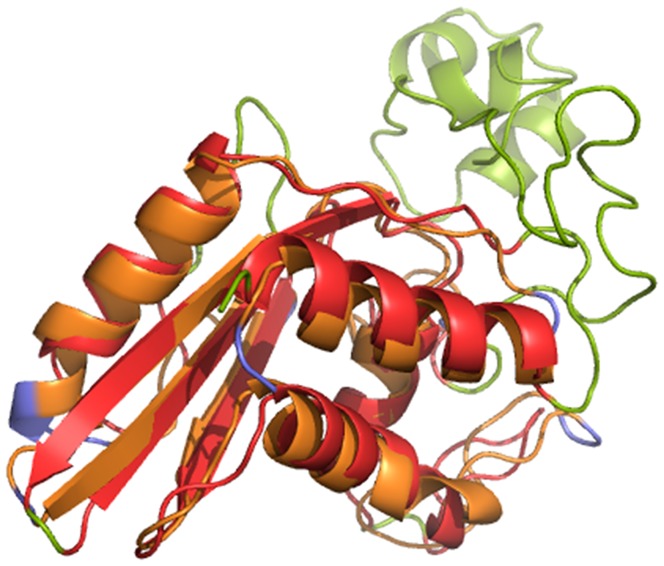
Superimposition of pseudechetoxin (PDB id: 1CFE, in green) and P14a (PDB id: 1B0F, in blue) and using TopMatch. The overlapping regions are colored red and orange for pseudechetoxin and P14a respectively. It is known that pseudechetoxin has elastase activity.

**Figure 3:**
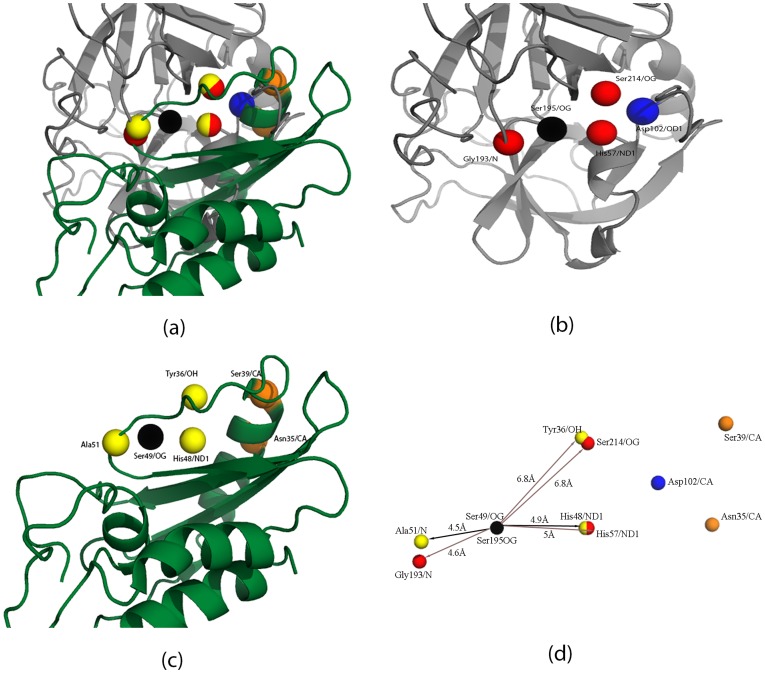
Superimposing proteins based on partial matches (Ser195, His57, Ser214 from PDBid:1B0F) and (Ser49, His48, Tyr36 from PDBid:1CFE). Ser195 from PDBid:1B0F and Ser49 from PDBid:1CFE coincide and is colored in black. (**a**) Superimposition of both proteins: Human neutrophilelastase (PDBid:1B0F) is in grey, and P14A (PDBid:1CFE) is in green. (**b**) Human neutrophil elastase (PDBid:1B0F) with active site atoms - Ser195/OG, His57/ND1, Ser214/OG, Gly193/N. (**c**) P14A protein (PDBid:1CFE) with predicted active site residues - Ser49/OG, His48/ND1, Tyr36/OH, Ala51/N. (**d**) Distance between pairs of residues in the partial matches. It can be seen that the Asp102 in PDBid:1B0F is close to Asn35 and Ser39 in PDBid:1CFE.

**Table 3:** Extending the partial match.

	a	b	c	d		
1B0F Motif	SER195	HIS57	SER214	GLY193		
1CFE motif	SER49	HIS48	TYR36	ALA51		
	**ab**	**ac**	**ad**	**bc**	**bd**	**cd**
**PDB**	**Pairwise Distances**					
1B0F	5	6.8	4.6	4.7	9.4	11
1CFE	4.9	6.8	4.5	5	9.1	10
	**Pairwise Potential difference**					
1B0F	53.7	105.7	−111.1	51.9	−164.9	−216.8
1CFE	15.3	88.6	−58.1	73.3	−73.4	−146.8

Spatial and electrostatic potential difference (PD) congruence in cognate pairs in the human neutrophil elastase (PDBid:1B0F) and the pathogenesis related P14A protein (PDBid:1CFE) for an extended set of four residues in the active site.

**Table 4:** Superimposing proteins based on partial matches (Ser195, His57, Ser214 from PDBid:1B0F) and (Ser49, His48, Tyr36 from PDBid:1CFE).

	Atom	Before			After		
	Coordinates	X	Y	Z	X	Y	Z
PDB: 1B0F (template)	Ser195/OG	64.4	57	53.8	0	0	0
	His57/ND1	63.3	54.8	58.2	5	0	0
	Ser214/OG	63.6	50.6	56.1	5	4.7	0
PDB: 1CFE(target)	Ser49/OG	8.8	−6.3	−4.8	0	0	0
	His48/ND1	9.2	−2.4	−1.8	4.9	0	0
	Tyr36/OH	13.7	−1.6	−3.7	4.7	5	0

After applying linear and rotational transformations on both proteins, Ser195 and Ser49 lie at the center of the coordinate axis, His57 and His48 lie on the -X-Y axis (Y = 0) and Ser214 and Tyr36 lie on the X–Y plane (Z = 0).

**Table 5:** Gain of potential congruence in L158E mutant of PBP-A.

	a		b		c		d		e									
1E25	Ser70/OG		Lys73/NZ		Ser130/OG		Lys234/NZ		Glu166/OE1									
2J7V	Ser61/OG		LYS64/NZ		Ser122/OG		LYS219/NZ		Leu158/CD2									
2J9O	Ser61/OG		Lys64/NZ		Ser122/OG		Lys219/NZ		Glu158/OE1									
1NZO	Ser44/OG		Lys47/NZ		Ser110/OG		Lys213/NZ		Leu153/CD2									
		**ab**		**ac**		**ad**	**ae**	**bc**		**bd**		**be**		**cd**		**ce**	**de**	
1E25	PD	−125.6		22.4		−189.1	310.7	148.1		−63.5		436.3		−211.5		288.2	499.7	
1E25	D	2.8		3.2		4.7	5.5	3.6		5.6		5		2.9		8	10	
2J7V	PD	−134.5		38.7		−135.6	−112.4	173.3		−1		22.1		−174.3		−151.1	23.2	
2J7V	D	3		3		4.4	7.1	3.1		5.4		5.8		2.9		8.7	10.1	
2J9O	PD	−162.3		−16.4		−198.3	247.7	146		−36		410		−181.9		264	445.9	
2J9O	D	2.7		3		4.5	4.5	3		5.3		4		2.8		6.7	8.6	
1NZO	PD	−241.6		−68.8		−277.9	−188.2	172.8		−36.2		53.5		−209.1		−119.4	89.7	
1NZO	D	3.1		4.2		6.3	6.5	5.1		6.7		5.5		2.7		10	12.1	

Pairwise potential difference (PD) and distance (D) between cognate pairs in a Class A Blase (PDBid:1E25), a PBP-5 protein (PDBid:1NZO), the wildtype PBP-A (PDBid:2J7V) and the L158E PBP-A mutant (PDBid:2J9O). The L158E PBP-A mutant has gains potential congruence with the Class A Blase, as compared to the wildtype PBP-A. Distances are in A. See Methods section for units of potential.

There have been several methods developed for identifying enzymes with a partial catalytic structure [47]–[49]. The use of ‘exact’ electrostatic properties in the search process at an early stage is a key innovation of DECAAF when compared to these methods. Other related methods have used binding energy and energy minimization considerations at a later stage of the search [24], [25], [27]. Although, the CLASP signature for any function comprises of a few residues, it implicitly encodes the surroundings. Potential congruence implies a favorable milieu for the particular function (hydrophilicity, polarity, presence in a cleft, etc.). Consequently, DECAAF filters out unfeasible configurations at a much lower computational cost.

**Figure 4:**
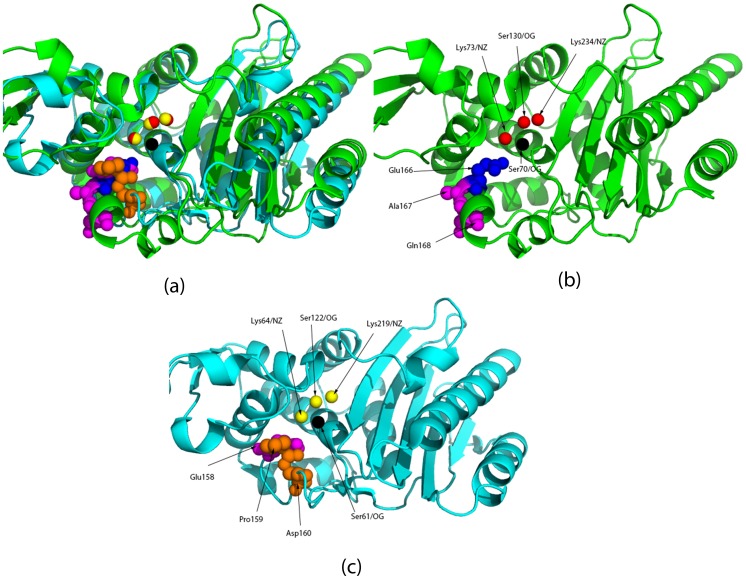
Superimposition of Class A Blase (PBDid:1E25 in green) and L158D PBP-A mutant (PDBid:2J9O in light blue) based on their partial matches - (Ser70/OG, Lys73NZ, Ser130/OG) and (Ser61/OG, Lys64/NZ, Ser122/OG) respectively. (**a**) It is seen that transformations aimed at superimposing the partial matches results in a good superimposition of the complete proteins. (**b**) Class A Blase (PBDid:1E25) - it can be seen that the Glu166/OE1 has no steric hindrance from the adjacent residues (Ala167 AND Gln168). (**c**) Mutant L158D PBP-A (PDBid:2J9O) - neighboring residues, Pro159 and Asp160, can be seen obstructing access to substrate.

**Table 6:** Atoms close to the Glu166/OE1 in Class A β-lactamase after superimposition of the partial motifs.

1E25			2J7V			2J9O			1NZO		
D	Residue	Atom	D	Residue	Atom	D	Residue	Atom	D	Residue	Atom
0	Glu166	OE1	1.6	Pro159	C	1.6	Pro159	C	1.4	LEU153	CD2
1.3	Glu166	CD	1.6	Pro159	CA	1.6	Pro159	CA	2.7	LEU153	CG
2.2	Glu166	OE2	1.8	Pro159	O	1.9	Glu158	OE1	2.8	HOH475	O
2.4	Glu166	CG	2.4	Leu158	CD1	1.9	Pro159	O			
2.7	Glu166	CB	2.7	Asp160	N	2.6	Asp160	N			
3	Glu166	CA	2.7	Pro159	N	2.7	Glu158	CA			
3.1	HOH2076	O	2.7	Leu158	CA	2.7	Pro159	N			
			2.8	Pro159	CB	2.8	Glu158	CB			
			2.9	Leu158	CB	2.8	Glu158	CD			
			3.1	Leu158	CG	2.9	Pro159	CB			
			3.2	Leu158	C	3.1	Glu158	C			
			3.4	HOH2146	O	3.3	HOH2234	O			

PDBid:1E25 is the Class A Blase, PDBid:2J7V is the wildtype PBP-A, PDBid:2J9O is the L158E PBP-A mutant and PDBid:1NZO is the PBP-5 protein. It can be seen that in the Class A Blase (PDBid:1E25) and PBP-5 there are no atoms within a radius of about 3 Å of Glu166/OE1. In the mutant L158E PBP, both Pro159 and Asp160 are spatially close to Glu158, and can be seen to be obstructing access to substrate (Fig. 4). Distances are in Å, and have a cut-off till the first water molecule is encountered.

**Figure 5:**
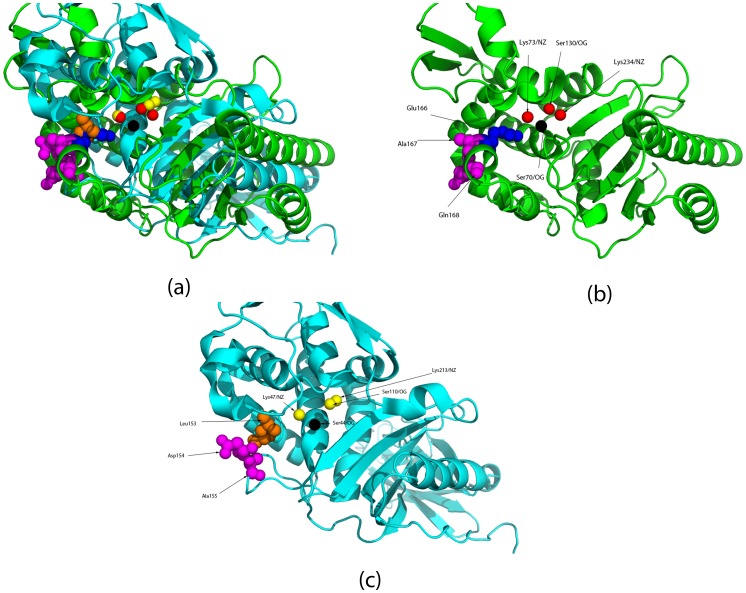
Superimposition of Class A Blase (PBDid:1E25 in green) and PBP-5 (PDBid:1NZO in light blue) based on their partial matches - (Ser70/OG, Lys73NZ, Ser130/OG) and (Ser44/OG, Lys47/NZ, Ser110/OG) respectively. (**a**) It is seen that the superimposition of PBP-5 and this Class A Blase is less significant than that with PBP-A. (**b**) Leu153 corresponds to the spatial location of Glu166, and it is seen that Asp154 and Ala155 do not restrict access to the catalytic cleft.

We have worked out the detailed steps in DECAAF for identifying an elastase-like protein in plants. Our motivation is to find an alternative for human neutrophil elastase (HNE) [50] in a chimeric enzyme that provides enhanced resistance to the Pierce’s disease causing Gram-negative pathogen *Xylella fastidiosa* [51]. The substitute should be preferably from an organism related to grapevines. P14A [52] from *Solanum lycopersicum* (tomato), a member of the PR-1 group of pathogenesis-related proteins [53], is obtained as a suitable candidate. The structural homology shared by P14A and a snake venom protein, which was previously demonstrated to be an elastase [54], suggests with increasing certitude that P14A might have elastase activity. Furthermore, protease function has also been associated with the pathogenesis related proteins [55]. We have also identified residues in P14A that do not match the corresponding residue in HNE, and thus predicts mutations in the P14A which should replicate the HNE scaffold. In case we do not find detectable activity in the wild type P14A, we believe that the identified P14A mutants might gain elastase function**,** Finally, we propose that a L153E mutant of PBP-5 from *E. coli* (PDBid:1NZO) is likely to show better results than the L158E mutant of PBP-A from *T. elongatus* (PDBid:2J9O), which achieved a 90-fold increase in β-lactamase activity. Such experimental validations will be the ultimate litmus test for the DECAAF flow.

## Results

We demonstrate our method (DECAAF) by working out the steps in selecting a elastase-like protein from a set of plant proteins. Human neutrophil elastase (HNE) is a serine protease that is present in the primary granules of polymorphonuclear neutrophils, the other two being proteinase 3 and cathepsin G [56]. The bactericidal properties of HNE have been exploited to design a anti-microbial protein that targets the outer-membrane of the bacteria [57]. HNE is combined with cecropinB, which lyses gram-negative bacteria, in this chimeric protein using a flexible linker and provides enhanced resistance to grapevines against the Gram-negative pathogen Xylella fastidiosa. A protein from a plant related to grapevines that has elastase activity, if found, can substitute HNE in the anti-microbial enzyme.

### 1 Selecting the Best Candidate

We chose the HNE protein with PDBid:1B0F as the template protein. The active site residues comprised of the following residues - Ser195, His57, Asp102, Ser214 and Gly193. The keyword search for ‘plants’ in http://www.pdb.org/was pruned for redundancy based on a 40% sequence similarity, and yielded 288 proteins (Table S1). From the active site residues (N = 5), we generated all possible motifs of size k < N (3, 4 and 5 in this case). An example motif le is shown in Fig. 1. By default each position in the match can be occupied by the same amino acid, i.e. we do not consider stereochemical equivalence at this stage.

Let us consider the case when the partial motifs have three residues - there are 10 such motifs (^5^_3_ = 10). M1 = (Ser195, His57, Ser214), M2 = (Ser195, His57, Gly193) … M10 = (Ser195, His57, Asp102). We ran each motif (M1, M2, … M10 ) on each protein in the target list, resulting in a score that quantifies the degree to which a motif exists in a protein based on spatial and electrostatic congruence [29]. Each run of a single motif on the list of 288 proteins took less than 30 mins on a simple workstation (2 GB Ram). Fig. 1 shows the best matches with M1 and M2.

We then compute a cumulative score from the runs for different motifs for each protein that reflects the likelihood of endowing elastase to that protein (Table 1). Manual inspection now chose the best protein from the reduced set of the proteins. The small number of proteins to be manually evaluated made this feasible. The best matching protein (PDBid:1DX6) is from *Torpedo californica* (Pacific electric ray), an organism not related to plants. The acetylcholinesterase protein is included in the set of proteins being queried since it has a plant alkaloid galanthamine bound in active site, and we used a keyword search for ‘plant’ in http://www.pdb.org/to obtain the query set of 288 proteins. The P14A (PDB id: 1CFE), the next best ranked protein, from *Solanum lycopersicum* (tomato) was therefore the desired target protein [52]. Table 2 shows the congruence of the partial motif (Ser195, His57, Ser214) from the HNE protein with the match from P14A.

P14A is a member of the PR-1 group of pathogenesis-related proteins [53]. While P14A has not been specifically known to have elastase activity, it is structurally homologous to a snake venom protein (Fig. 2) which was previously demonstrated to be an elastase [54]. Pathogenesis related proteins are known to possess proteolytic function [55]. Furthermore, it has been noted that “His48, Ser49 and His93 are in close proximity, so that the conservation of this group of residues could be taken as an indication of it being important as an active site in P14a” [52]. His48 and Ser49 are the residues predicted by DECAAF to be a part of the catalytic scaffold (Table 2 and Fig. 3).

A similar analysis using partial motifs of size k = 4 and k = 5 was done. We have not been able to design a reasonable algorithm for comparing scores with different motif lengths. The P14A protein does not have a good match when the input scaffold includes Asp102. Hence, any motif figuring the Asp102 was not found in P14A with significant congruence. We discuss this aspect subsequently.

### 2 Improving Matches through Stereochemical Equivalence

We extended the matches by allowing stereochemically equivalent residues to match in a motif. While stereochemical equivalence can be hardcoded for amino acids with similar properties, there are instances where residues with different properties occupy the same sequence and spatial location, and perform the same functionality. A well-known example is the equivalence of Ser130 and Tyr150 in Class A and C - lactamases respectively [58]. In the motif (M1 = Ser195, His57, Ser214), we allowed the position Ser214 to be matched by either a Ser or a Tyr. Table 2 shows that Tyr36 has a much better spatial orientation than the Ser120 in the P14A protein, although the potential differences are almost similar. Similarly extending the motif length (n = 4) allowed us to match Gly193 in the HNE protein to Ala51 in the P14A protein (Table 3 and Fig. 3). Gly and Ala both have non-polar, aliphatic R groups.

### 3 Superimposing the Partial Scaffolds

As mentioned previously, the P14A protein does not have a good match when the input scaffold includes Asp102. Although, unconventional serine proteases with variation in the catalytic triad have been observed in many cases, it is possible that P14A is not one them [59]. In such a scenario, we would have to resort to directed evolution techniques to impart elastase function to the protein. Table 4 shows the coordinates of the atoms before and after the transformations when the partial match - (Ser195/OG, His57/ND1, Ser214/OG, Gly193/N - PDBid:1B0F) and (Ser49/OG, His48/ND1, Tyr36/OH, Ala51/N - PDBid:1CFE) - are superimposed. Note, that the superimposition algorithm uses the first three residues in the match. Fig. 3 shows the superimposed proteins. It can be seen that the pair (Gly193/N, Ala51/N), although not used for superimposition, are spatially close. The spatial position of the Asp102 in the HNE is in the vicinity of Asn35 and Ser39 in the P14A protein. Hence, it is possible that the Asn35Asp or Ser39Asp mutant might gain elastase function.

### 4 Analyzing Results of Directed Evolution of Penicillin Binding Proteins to Enhance β-lactamase Activity

The technique for superimposing the active sites of proteins based on the partial matches has been applied to analyze the lower than expected gain in β-lactamase (Blase) activity of a penicillin binding protein (PBP) using site directed mutations [43]. Class A Blases are differentiated from other classes of Blases (C and D) by virtue of the presence of a loop, which houses the critical Glu166 [46]. PBP-A from *T. elongatus* possesses a similar loop, and sequence alignment suggested that Leu158 occupies the position corresponding to Glu166 (of the Blase) in this protein. However, the mutant L158E showed a meager 50-fold gain in activity as compared to the wildtype. This gain in activity is corroborated by CLASP analysis of the mutant active site, which gains potential congruence in cognate pairs as compared to the Class A Blase catalytic residues (Table 5). This electrostatic congruence was missing in the wildtype (Table 5).

The superimposition of the two proteins, Class A Blase (PB Did:1E25) and L158E PBP mutant (PDBid:2J9O) based on their partial matches - (Ser70/OG, Lys73NZ, Ser130/OG) and (Ser61/OG, Lys64/NZ, Ser122/OG) respectively, suggested that the residues adjacent to Glu158 in the mutant protein (Pro159 and Asp160) are responsible for hindering substrate access (Fig. 4). Pro159 and Asp160 are spatially close to Leu158/Glu158 in the wildtype/mutant, whereas in the Class A Blase there are no residues within 3 Å of Glu166 (Table 6). Several combinations of mutations failed to improve the activity any more than 90-fold. Pro159 was conserved in all mutants, and hence possibly continued to deny access to substrate in the mutations.

Subsequently, we chose PBP-5 from *E. Coli*, which is also known to have a similar loop like the Class A Blases and PBP-A [45]. It has been noted that a His151 occupies the same spatial position as Glu166, and is possible a good target for mutation – “spatial equivalence of carbonyl group of His-151 in PBP 5 with the carboxyl group of Glu-166 in TEM-1” [60]. However, Leu153 showed up as the best candidate for mimicking Glu166 when we superimposed the Class A Blase and the PBP-5 (PDBid:1NZO) based on partial matches. Furthermore, the adjacent residues to Leu153 do not impede the substrate (Table 6 and Fig. 5). We hypothesize that a mutational study similar to the L158E of PBP-A, if applied to PBP-5 to generate L153E, might provide greater success in replicating Blase enzymatic efficiency in PBPs.

## Discussion

Mutations resulting from the innate inaccuracies of biological processes and environmental factors accrue over millions of years and are a key determinant of evolution. Rapid technological advances in the last few decades have allowed enzyme engineers to apply similar mutation generating methods, and shrunk the timescales for evolving new enzymes to a few months [1]–[5]. Directed evolution is the generic term applied to experiments that mimic and accelerate natural evolution [13]–[20], [61]. Several groups have reviewed the current developments in directed evolution in detail [62]–[65].

Promiscuity, the catalysis of reactions distinct from the one the protein has evolved to perform, is another characteristic feature of enzymes that has shaped the evolution of primitive cells [33], [66], [67]. Gene duplication unshackled such ‘leaky’/’messy’ domains from selection pressures, and subsequent specialization culminated into new enzymes [34], [35], [68]. Identifying pre-existing scaffolds in proteins is a critical step as it implies better odds of success when attempting to endow catalytic activity to proteins [24]–[28].

The current work has tried to exploit this prospect of a promiscuous active site to serve as a pre-existing scaffold for directed evolution. Previously, we have defined a quantitative measure of promiscuity in a wide range of proteins [32]. The spatial and electrostatic congruence of the active site residues in a heme cytochrome C peroxidase and a Zn^2+^ carboxypeptidase was vindicated by the fact that carboxypeptidase is known to gain pyruvate oxidase catalysis abilities on the replacement of Zn^2+^ with Cu^2+^ [69]. This germinated the idea of defining a generic methodology that would seek an ‘incubator’ protein for any enzymatic activity with known 3D structure and active site residues.

We present a computational method that selects a protein which has a significant match with a desired catalytic scaffold - ***D***irected ***e***volution using ***C***LASP: ***a***n ***a***utomated ***f***low (DECAAF). Barring the rare chance of finding a protein matching the complete motif, the problem at hand is to find a protein with the best partial matches. This has been solved using heuristics that generates a library of partial motifs and results in a cumulative score reflecting the likelihood of endowing the desired function to that protein. The intractability of the problem is evident, and the many methods are inclined to remain in a local minima [70]. Hence the final step might involve manual screening of a few best matching proteins. Expert eyes can also figure out other supporting facts - for example, it might be known that a related protein from the same superfamily possesses the function, instilling further confidence before one embarks on laborious mutational work.

The possibility that the selected protein does not possess the activity, despite the presence of a subset of the catalytic motif in its structure, leads to the next logical question - can one predict mutations that would bestow function? We have addressed this requirement by superimposing the template and target proteins based on the partial matches, and identified target residues that are in the vicinity of ‘unmatched’ residues of the template protein. Such residues can be the target of site-directed methods [18]–[20].

This superimposition technique has been used to analyze the results of an experiment intended to transform a penicillin binding protein (PBP) into a serine β -lactamase (Blase). PBPs are involved in the synthesis and remodeling of bacterial cell wall [71]. Owing to the lack of a counterpart in mammalian organisms, PBPs are the target of the β lactam family of antibiotics (penicillin, ampicillin, etc.) which are structurally homologous to the D-ala-D-ala moiety present on peptidoglycan precursors. These drugs acylate the nucleophilic serine residue and, unlike the peptide substrates, form long-lived complexes. Blases, which share three highly conserved motifs with PBPs (the SXXK tetrad, the SXN triad, and KTG triad) are similarly acetylated, but have evolved to gain significantly higher rates of deacylation resulting in the regeneration of the original uncomplexed enzyme [37]–[39]. This sets up the scene for a perfect directed evolution experiment - what are the mutations needed to transform a PBP into a Blase? However, this task has proved to be surprisingly difficult [42], [43].

The serine Blases are divided into three classes (A, C and D) based on sequence homology [72]. Of these, the Class A Blases have evolved to use a Glu-X-X-Asn motif present on a loop (the loop) for catalysis [46]. PBP-A from *T. elongatus*, a thermophilic unicellular rod shaped cyanobacterium, possesses a similar loop, and sequence alignment suggested that Leu158 occupies the position corresponding to Glu166 in this protein [73]. However, the L158E PBP-A mutant could only achieve a meager 90-fold gain of activity, even when aided with other adjacent mutations [43]. This was ‘puzzling’ since ‘in the L158E mutant, all the catalytic residues and the interactions characteristic of β-lactamases seem to be present’ [43].

We have corroborated that the electrostatic properties of the active site residues in the L158E mutant now mirrors the Class A Blase catalytic site properties, explaining the increase in activity. Moreover, superimposition of the reactive atoms of the catalytic residues in both the protein provides a possible explanation of the modest gains - the residues adjacent to Glu158 in the mutant PBP-A (Pro159 and Asp160) is seen to be restricting access to the catalytic cleft, in stark contrast to the Class A Blase. Since Pro159 was conserved in all of the mutants, it might be a key determinant for the less than expected gain of activity.

Next, we chose PBP-5 from *E. coli* since it possesses a loop similar to Class A Blases and PBP-A [45]. PBP-5 helps in maintaining the normal morphology of the cell and provides resistance to β-lactams antibiotics [74]–[76]. While it has been commented that His151 is the residue that corresponds to the Glu166 in Class A Blase [60], we noted that Leu153 is a better candidate after superimposition of the proteins using the technique detailed in this work. Also, unlike in PBP-A, the adjacent residues to Leu153 in PBP-5 have a conformation similar to that in Class A Blases, and seem to provide unrestricted access of substrate to the catalytic site. We hypothesize that the L153E PBP-5 mutant might achieve better gain of function as compared to the L158E mutant(s).

The use of ‘exact’ electrostatic properties in the search process at an early stage is a key innovation of DECAAF when compared to similar methods seeking partial catalytic structure [47]–[49]. Pruning based on electrostatic properties reduces considerable false positives compared to purely 3D matching methods. A few methods have used binding energy and energy minimization considerations, albeit at a later stage of the search [24], [25], [27]. Electrostatic congruence of a few active site residues implies a favorable milieu for the catalytic activity and thus encodes residues in the close vicinity as well. This allows DECAAF to prune unfeasible configurations at a much lower computational cost.

We describe in the details the steps (Fig. 1) involved in choosing a plant protein to substitute human neutrophil elastase (HNE) [50] in a chimeric anti-microbial enzyme designed to bolster the innate immune defense system in grapevines [51]. The flow identifies P14A [52] from *Solanum lycopersicum* (tomato) as a very significant match (Tables 1, 2, 3). P14A is a member of the PR-1 group of pathogenesis-related proteins [53]. While P14A has not been specifically known to have elastase activity, it is structurally homologous to a snake venom protein (Fig. 2), previously demonstrated to be an elastase [54]. Interestingly, the complete scaffold of the HNE does not exist in the P14A protein. While Ser195, His57, Ser214 and Gly193 from the input motif has an highly matching scaffold in P14A (Fig. 3), the spatial position of the Asp102 in the HNE is close to Asn35 and a Ser39 in the P14A protein when the proteins were superimposed. We intend to express this protein and test for its elastase activity. In case we do not find detectable activity, we believe that the Asn35Asp or the Ser39Asp P14A mutant might gain elastase function and thus validate the proposed rational design flow DECAAF.

## Methods

### 1 Choosing a Consensus Group of Proteins

We start with a template protein with known 3D structure and a set of active site residues

(Φ_residues,_ | Φ_residues_ | = N) (Equation 1).

Our goal is to select the best protein from a set of proteins (Φ_proteins_ **, |** Φ_proteins_ **| = **M) with known 3D structures (Equation 2), that has a domain in its structure which is significantly congruous to Φ_residues_

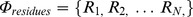
(1)

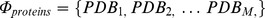
(2)We first create partial motifs of size k from **Φ_residues_**, for k = >3, 4…N (Equation 3). Let us denote the number of elements in ^n^C_k_ by n.
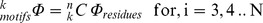
(3)CLASP analysis of each motif of size k results in n scores for each protein Pi (CLASPScorei). We combine this score into a cumulative score Score (PDBki ) (Equation 4).



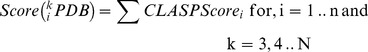
(4)Now, for each k, we obtained the sorted set of scores for each protein (Equation 5).
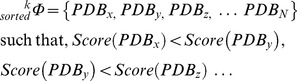
(5)


At this stage, we manually screen the proteins which occur in the top matches (**PDB_x_, PDB_y_, PDB_z_ …**) in Φ^k^_sorted_ for each k, and decide the best possible candidate.

### 2 Superimposing Proteins Based on Partial Matches and Predicting Mutations

We apply transformations on the template and target proteins such that the partial matches overlap. The superimposition algorithm takes the first three atoms of the partial match from both the template and target proteins - let us refer to them as (A,B,C) and (P,Q,R). We apply both linear and rotational transformations on both the proteins such that A and P are at the center of the coordinate axis (coordinates = [0,0,0]), B and Q lies on the X-Y axis (i.e. Y coordinate is 0) and C and R lie on the X-Y plane (i.e. Z coordinate is 0). In short, after the transformations, (A,B,C) and (P,Q,R) both lie on the same plane (Z = 0) such that A and P are at the center, and B and Q lie on the x-y axis. Once the two structures are superimposed, we proceed to find the residues in the target protein which are spatially close to the unmatched residues in the template protein. In case the wild type protein does not have the desired functions, mutating these residues might bestow function.

### 3 Tools

Adaptive Poisson-Boltzmann Solver (APBS) and PDB2PQR packages were used to calculate the potential difference between the reactive atoms of the corresponding proteins [77], [78]. The APBS parameters were set as follows - solute dielectric: 2, solvent dielectric: 78, solvent probe radius: 1.4 A, temperature: 298 K and zero ionic strength. APBS writes out the electrostatic potential in dimensionless units of kT/e where k is Boltzmann’s constant, T is the temperature in K and e is the charge of an electron. We extensively integrated and used the freely available BioPerl [79] modules and Emboss [80] tools. 3D structure was aligned by the TopMatch server [81]. All protein structures were rendered by PyMol (http://www.pymol.org/).

## Supporting Information

Table S1 **Target set of 288 proteins.** The keyword search for ‘plants’ in http://www.pdb.org/was pruned for redundancy based on a 40% sequence similarity, and yielded 288 proteins.
(PDF)
