# Supplementary material for: An Automated Flow for Directed Evolution Based on Detection of Promiscuous Scaffolds Using Spatial and Electrostatic Properties of Catalytic Residues
Source: PLoS One. 2012 Jul 11;7(7):e40408. doi: 10.1371/journal.pone.0040408 (PMC3394801; doi:10.1371/journal.pone.0040408)
Supplement: Table S1 — Target set of 288 proteins. The keyword search for ‘plants’ in http://www.pdb.org/was pruned for redundancy based on a 40% sequence similarity, and yielded 288 proteins. (PDF) [file pone.0040408.s001.pdf]

Table S1: The keyword search for 'plants' in http://www.pdb.org/ was pruned for redundancy based on a 40% sequence similarity, and yielded 288 proteins:
2VY0 1QMG 1CFE 2JEM 3HGO 2O7R 1DX6 1IYN 1YHZ 1USW 2HOR 1SQD 1GP6 1T3K 3I6S 1XKL 2EFC 3H2G 1NAR 1IEQ 1F2N 2CC0 3E5N 2J5C 2C71 1N10 1QRR 2PMP 1BGP 1N9L 2BGL 2CJP 1CWU 1ABR 2C7Y 1AVB 1OYV 2CJL 1AIR 1AZD 1B8G 1ZXZ 1OCK 2C1C 2R60 2BMO 1UV4 1S2O 2BHW 1QCX 1Z98 2ACV 1L3A 2EFJ 1BT3 1E1E 3KAL 2D5H 1YQD 1MVL 1SE9 1S21 2O98 1OGQ 1WFJ 1BK7 1W1O 1AQ0 1V2B 2K6H 2QSU 1CNV 1CWP 1CAU 2BIH 2DRE 1EJB 2IDR 1OXW 1W1Z 1EYP 1ZU2 2D3A 1Y5R 2DT4 1SM7 1H2O 2OPC 1V0L 1J4S 2R2G 1DK5 4SBV 1X8Z 1D8U 1SUI 1PC8 1S28 1FXZ 2JDA 1OPO 1R5E 1W77 1WID 2OKY 1AUY 1QN4 1JPC 1G28 1S6D 2CLK 1FP2 1MZ4 1F7S 1A0K 1GWC 1T97 1JLJ 1BW3 1AF5 1ASO 1AW9 1B5F 1BBG 1BEA 1BI5 1BK8 1BT0 1C01 1CCR 1CE3 1CQ4 1CTJ 1DCF 1DJ2 1DKC 1DLY 1DXL 1E2W 1E5X 1EN2 1EQK 1F56 1FB6 1FC6 1FCT 1GCC 1GME 1GOX 1GPS 1GQ8 1H20 1HPC 1HSS 1HTX 1HYP 1IRZ 1IUQ 1IYM 1J6Y 1J93 1JER 1JH6 1JKN 1JU2 1JXC 1JY5 1L3P 1LED 1LLN 1LU0 1M9L 1MHM 1MMC 1MZK 1N4N 1N7H 1OQP 1P8B 1P9G 1PJU 1PLC 1PSY 1PXY 1PYV 1QGN 1R8N 1RH9 1RJJ 1RL0 1RP0 1RPX 1S57 1SEZ 1SS3 1T0G 1T1H 1TH5 1TIZ 1TQ1 1TUK 1UAS 1UHA 1UHT 1UL5 1UMZ 1UUY 1V31 1V32 1V5N 1V9X 1VEK 1VG5 1WE9 1WEE 1WEO 1WEW 1WF9 1WG2 1WGP 1WH5 1WHS 1WIJ 1WJ2 1WJJ 1WVK 1X1N 1X32 1XDX 1XMT 1XO8 1XOY 1YEL 1YP2 1Z7W 1ZLP 2A3L 2AAK 2AAO 2AB9 2AC1 2AHN 2AJE 2AKJ 2APJ 2AYD 2B1M 2B38 2B7U 2BGH 2BGS 2BRJ 2C4B 2CDQ 2CJJ 2CND 2DCP 2DCQ 2DYD 2E1V 2E62 2E7P 2EBI 2F82 2FJ8 2G0Q 2GCU 2HJ3 2ICY 2J3J 2JON 2KAK 2KAN 2KD0 2KMW 2O66 2O7S 2OG2 2OQQ 2P9O 2PU9 2Q41 2Q49 2V6G 2VY2 3COB 3DM0 3DSK
